# Heroin Contaminated with Fentanyl Dramatically Enhances Brain Hypoxia and Induces Brain Hypothermia

**DOI:** 10.1523/ENEURO.0323-17.2017

**Published:** 2017-10-30

**Authors:** Ernesto Solis, Keaton T. Cameron-Burr, Eugene A. Kiyatkin

**Affiliations:** Behavioral Neuroscience Branch, National Institute on Drug Abuse – Intramural Research Program, National Institutes of Health, Baltimore, MD 21224

**Keywords:** brain temperature, metabolic activation, opioid, oxygen, vasoconstriction, vasodilation

## Abstract

While opioid abuse is an established medical and public health issue, the increased availability of highly potent synthetic opioids, such as fentanyl, has given rise to acute health complications, including a comatose state and death during drug overdose. Since respiratory depression that leads to acute hypoxia is the most dangerous complication of opioid drug use, we examined the effects of intravenous heroin and heroin contaminated with 10% fentanyl on oxygen levels in the nucleus accumbens (NAc) monitored using high-speed amperometry in freely moving rats. Additionally, we examined the effects of heroin, fentanyl, and their mixture on locomotion and temperatures in the NAc, temporal muscle, and skin. Both fentanyl and heroin at human-relevant doses (400 and 40 μg/kg, respectively) induced rapid, strong and transient decreases in NAc oxygen, indicative of brain hypoxia. When the heroin-fentanyl mixture was injected, the NAc hypoxic response was greatly potentiated in its duration, suggesting sustained hypoxia. In contrast to modest, monophasic brain temperature increases caused by heroin alone, the heroin-fentanyl mixture induced a biphasic temperature response, with a prominent postinjection decrease resulting from peripheral vasodilation. This hypothermic effect, albeit much smaller and more transient, was typical of fentanyl injected alone. Our findings indicate that accidental use of fentanyl instead of heroin, or even a relatively minor contamination of “street heroin” with fentanyl, poses great danger for acute health complications, including a comatose state and death.

## Visual Abstract

**Figure:**
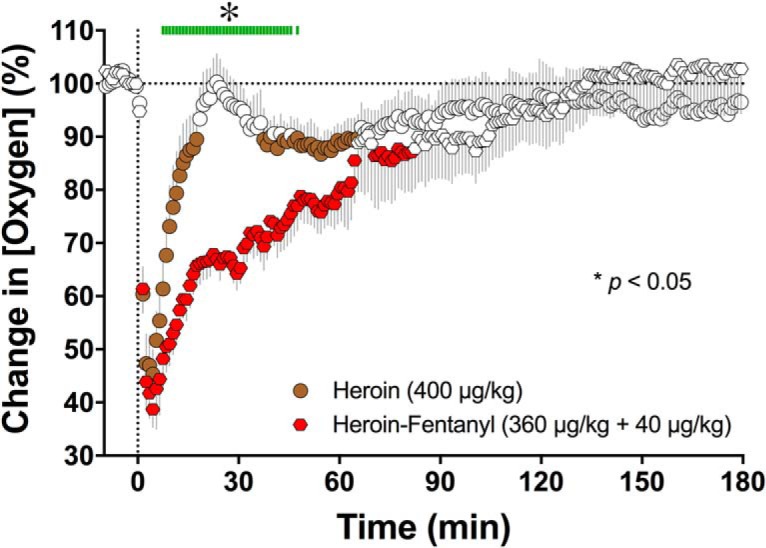

## Significance Statement

Respiratory depression with subsequent acute hypoxia is the leading cause of lethality induced by intravenous heroin at high doses. This effect may be enhanced when the heroin consumed is contaminated with fentanyl, a much more potent opioid drug. By using high-speed amperometry with oxygen sensors in freely-moving rats, we show that both fentanyl and heroin at human-relevant doses induce rapid, strong but transient decreases in NAc extracellular oxygen levels. This brain hypoxic effect was greatly enhanced when rats received a heroin dose laced with 10% of fentanyl. Our findings indicate that the accidental use of fentanyl instead of heroin, or even a relatively minor contamination of “street heroin” with fentanyl, poses great risk for acute health complications, including a comatose state and death.

## Introduction

“Street heroin” may often be contaminated with fentanyl, a synthetic opioid drug 20–40× as potent as heroin (Wade et al., 2015). Due to fentanyl’s increased potency, intake of heroin-fentanyl mixtures may result in serious health complications, including a comatose state and lethality (Compton et al., 2016; Suzuki and El-Haddad, 2017). Both drugs induce respiratory depression (Yeadon and Kitchen, 1989; Dahan et al., 2005; Pattinson, 2008) that results in systemic hypoxia, placing individuals consuming heroin contaminated with fentanyl at risk for acute disturbances in neural function and possible lethality.

In this study, we examined the effects of heroin contaminated with fentanyl on brain oxygen levels directly assessed by high-speed amperometry with Pt-Ir oxygen sensors. This technology, recently developed in our lab, allows for the direct evaluation of rapid fluctuations in brain oxygen levels, a critical homeostatic parameter inaccessible before the advent of such technology. Our experimental paradigm allows us to monitor brain oxygen levels following stress- and cue-free intravenous drug injections in awake, freely moving rats and determine the effects of drugs of abuse in the absence of confounding variables such as anesthesia. Using this novel technology, we recently examined physiological fluctuations in brain oxygen levels and established their relationships with metabolic neural activation and local vascular response (Solis et al., 2017a). We also showed that both heroin and fentanyl used within the range of possible human consumption induce rapid, dose-dependent decreases in extracellular oxygen levels in the nucleus accumbens (NAc) of freely moving rats (Solis et al., 2017b,c). By monitoring oxygen in the subcutaneous space, a densely vascularized site with no metabolic activity, we proved that the decrease in NAc oxygen results from decreased blood oxygen levels due to heroin- and fentanyl-induced respiratory depression.

Our oxygen recordings were supplemented by monitoring temperature in the brain, temporal muscle, and skin. While brain temperature by itself is an important homeostatic parameter, the three-point temperature recording paradigm used in this study allowed us to determine the basic physiological mechanisms underlying drug-induced brain temperature responses: changes in intrabrain heat production due to metabolic brain activation/inhibition and changes in heat loss/retention due to changes in skin vascular tone (Kiyatkin, 2010). These data were used to examine the relationships between drug-induced brain oxygen changes, metabolic neural activation, and the vascular response. Like our previous work, we chose the NAc, a brain area involved in mediating the reinforcing properties of drugs (Wise and Bozarth, 1987; Di Chiara, 2002; Badiani et al., 2011) as a representative brain structure for oxygen and brain temperature recordings.

Here, we sought to mirror a real-world scenario in which an individual who believes he or she is consuming a standard dose of heroin is actually consuming a heroin sample laced with a smaller amount of fentanyl. We compared the effects of a control dose of heroin (400 μg/kg) and this dose contaminated with 10% fentanyl, which gives rise to a heroin-fentanyl mixture containing 360 μg/kg of heroin and 40 μg/kg of fentanyl. Both 400 μg/kg of heroin and 40 μg/kg of fentanyl are much lower than the LD50 assessed in rats [15–20 mg/kg (Gable, 2004; Strandberg et al., 2006); 1–3 mg/kg (von Gunten et al., 2010), respectively] and are within the range of consumption of experienced drug users (see erowid.org). No data exists in the literature on the effects of these drugs on brain oxygen levels and brain temperature when taken in combination.

## Materials and Methods

### Subjects

A total of 25 male Long-Evans rats (Charles River Laboratories) weighing 460 ± 40 g at the time of surgery were used in this study. Rats were individually housed in a climate-controlled animal colony maintained on a 12/12 h light/dark cycle (lights on at 6 A.M.) with food and water freely available. All procedures were approved by the National Institute on Drug Abuse Intramural Research Program (NIDA-IRP) Animal Care and Use Committee and complied with the Guide for the Care and Use of Laboratory Animals (National Institutes of Health, Publication 865-23). Maximal care was taken to minimize the number of experimental animals and avoid any possible discomfort at all stages of the study.

### Overview

This study describes the results of two experiments conducted in awake, freely-moving rats. In the first, thermorecording experiment (*n* = 12 rats), we examined the effects of iv fentanyl (40 μg/kg), heroin (400 μg/kg), and their mixture (360 μg/kg heroin + 40 μg/kg fentanyl) on temperatures in the NAc, temporal muscle, and skin as well as locomotor activity. In the second, electrochemical experiment (*n* = 13 rats), we examined the effects of fentanyl (40 μg/kg), heroin (400 μg/kg), and their mixture (360 μg/kg heroin + 40 μg/kg fentanyl) on extracellular levels of oxygen in the NAc.

### Surgical preparations

Surgical procedures for electrochemical experiments are described in detail elsewhere (Kiyatkin and Lenoir, 2012; Solis et al., 2017a). In brief, under general anesthesia (Equithesin, sodium pentobarbital and chloral hydrate mixture), each rat was unilaterally implanted with a BASi guide cannula (Bioanalytical Systems) into which an electrochemical sensor was later inserted. The target locations for the right NAc shell were: anterior-posterior, +1.2 mm; medial-lateral, +0.8 mm; and dorsal-ventral, +7.3 mm from the skull surface according to the rat brain atlas (Paxinos and Watson, 1998). The guide cannula was secured with dental acrylic in a head mount anchored to the skull. When not in use, stainless steel obdurators were inserted into the cannulas to prevent occlusion. During the same surgical procedure, rats were also implanted with a chronic jugular catheter, which ran subcutaneously to the head mount and was secured to the same assembly. Rats were allowed a minimum of 5 d of postoperative recovery and at least three daily habituation sessions (∼6 h each) to the recording environment before experimentation; jugular catheters were flushed daily with 0.2 ml heparinized saline to maintain patency.

Surgical procedures for thermorecording experiments have been described in detail elsewhere (Kiyatkin et al., 2016). In brief, under the same anesthesia, rats were implanted with a jugular catheter and three copper-constantan thermocouple electrodes in the NAc shell (anterior-posterior, +1.2 mm; medial-lateral, +0.8 mm, depth 7.3 mm below the skull surface), temporal muscle, and subcutaneously along the nasal ridge with the tip ∼15 mm anterior to the bregma. The probes were secured with dental acrylic to the three stainless steel screws threaded into the skull. In this experiment, we also measured drug-induced changes in locomotor activity using four infrared motion detectors (Med Associates) as previously described (Brown and Kiyatkin, 2004).

### Electrochemical detection of oxygen

For direct assessment of fentanyl-induced changes in brain oxygen, we used commercially produced oxygen sensors (Model 7002-02; Pinnacle Technology). Each sensor consists of an epoxy-sheathed disk electrode that is grounded to a fine surface using a diamond-lapping disk. The sensors are prepared from Pt-Ir wire 180 μm in diameter, with a sensing area of 0.025 mm^2^ at the sensor’s tip. The active electrode is incorporated with an integrated Ag/AgCl reference electrode. Dissolved oxygen is reduced on the active surface of these sensors, which is held at a stable potential of −0.6 V versus the reference electrode, producing an amperometric current. The current from the sensor is relayed to a computer via a potentiostat (Model 3104; Pinnacle Technology) and recorded at 1-s intervals, using the PAL 8100: Biosensors legacy software (version 1.5.0; Pinnacle Technology).

Oxygen sensors were calibrated at 37°C by the manufacturer (Pinnacle Technology) according to a standard protocol described elsewhere (Bolger et al., 2011). The sensors produced incremental current rises with increases in oxygen concentration within the wide range of previously reported brain oxygen concentrations (0–50 μM). Substrate sensitivity of oxygen sensors varied from 1.1–1.9 nA/1 μM (mean = 1.43 nA/1 μM). Oxygen sensors were also tested by the manufacturer for their selectivity toward other electroactive substances, including dopamine (0.4 μM) and ascorbate (250 μM), none of which had significant effects on reduction currents.

These sensors were previously used for assessment of physiological fluctuations in NAc oxygen (Solis et al., 2017a); this publication contains additional details on the use of this technology in awake, freely moving rats.

### Experimental procedures

Temperature recordings were conducted during three 6- to 8-h sessions, during which the rats received single iv injections of fentanyl (fentanyl citrate injection 50-μg/ml saline solution; Hospira; 40 μg/kg), heroin (Diamorphine HCl, obtained from the NIDA-IRP Pharmacy; 400 μg/kg dissolved in saline), and a freshly prepared mixture of heroin and fentanyl (360 and 40 μg/kg, respectively). All drug injections were conducted ∼2 h after the session start while the rats were under quiet resting conditions and temperatures had stabilized at their basal levels. Recordings continued for at least 4 h after drug injections. Each treatment session was followed by one drug-free day. Injections of heroin and the heroin-fentanyl mixture were counterbalanced.

Electrochemical recordings were performed in two groups of rats, which underwent ∼2 h of habituation to the recording environment. In the first group, the rats received a single injection of fentanyl (40 μg/kg). In the second group, recordings were conducted during two sessions (with one drug-free day in between) and each rat received both heroin (400 μg/kg) and a heroin-fentanyl mixture (360 and 40 μg/kg, respectively). The order of drug presentation was counterbalanced. At the beginning of each session, rats were minimally anesthetized (<2 min) with isoflurane and the sensor was inserted into the NAc. The rat was placed in the testing chamber and the sensor was connected to the potentiostat via an electrically shielded flexible cable and a multichannel electrical swivel. The injection port of the jugular catheter on the head mount was connected to a plastic catheter extension, which allows stress- and cue-free drug delivery from outside the cage. Testing began ∼120 min after insertion of the sensor, when baseline currents had stabilized.

At the end of experimentation, rats were deeply anesthetized with Equithesin (1.0 ml for 2 min, i.v.) and isoflurane and decapitated. Then, the brain was removed, stored in 10% formalin, and sectioned for verification of sensor placements using a rat stereotaxic atlas (Paxinos and Watson, 1998); the brain sections were also assessed for possible gross anatomic damage.

### Data analysis

Temperatures were sampled at 10-s time bins and later analyzed at two time resolutions (slow, with 1- and 2-min time bins, and rapid, with 10-s bins) to determine drug-induced changes in absolute temperature in each recording location, relative temperature, and NAc-muscle and skin-muscle temperature differentials (a difference between relative temperature changes in the corresponding locations). As described in our previous studies (for review, see Kiyatkin, 2010), by simultaneously recording temperature from these three locations, it is possible to assess the effect of a physiological or drug stimulus on intrabrain heat production due to metabolic brain activation and heat loss or retention due to changes in skin vascular tone (vasoconstriction/vasodilation). Data were statistically analyzed with one- and two-way repeated measures ANOVAs and significant ANOVA results were followed up with Fisher’s PLSD *post hoc* tests.

Electrochemical data were sampled at 1 Hz (i.e., mean current over 1 s) using the PAL software utility (version 1.5.0; Pinnacle Technology) and analyzed with two time resolutions (slow, with 1-min time bins, and rapid, with 4-s bins). Electrochemical data were first analyzed as raw currents. Because each of the individual sensors differed slightly in background current and substrate sensitivity *in vitro*, currents were then transformed into concentrations and are represented as relative concentration changes, taking a pre-stimulus baseline current as 0. Due to between-sensor variability in oxygen sensitivity, we also analyzed drug effects as percentage with respect to baseline (=100%). One-way and two-way repeated measure ANOVAs (followed by Fisher PLSD *post hoc* tests) were used to evaluate the statistical significance of drug-induced changes in oxygen levels. We also used time-dependent correlation analyses to examine the relationships between oxygen, brain temperature, and temperature differentials.

## Results

Data presented in this report were obtained in rats with artifact-free neurochemical and temperature recordings. In each rat, the locations of brain sensors were histologically verified to be located within the NAc shell.

### Effects of fentanyl

**Figure 1.**
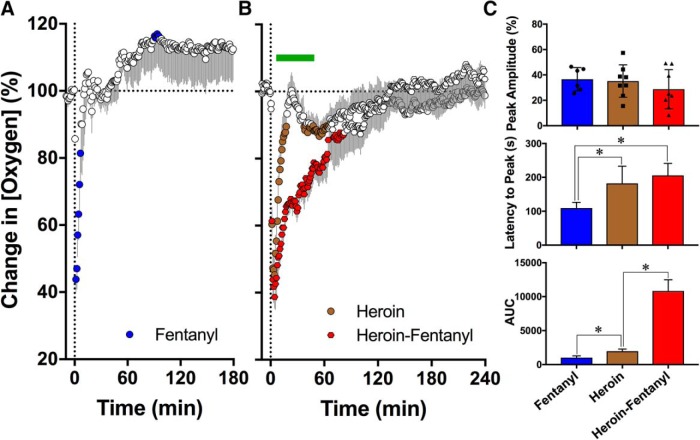
Changes in NAc oxygen levels (normalized as percentage vs baseline = 100%) induced by intravenous injections of fentanyl (40 µg/kg; ***A***), heroin (400 µg/kg; ***B***), and their mixture (40 µg/kg fentanyl + 360 µg/kg heroin; ***B***) in awake, freely moving rats. Changes in NAc oxygen were significant for each of the three treatments. Values significantly different from baseline (*p* < 0.05; horizontal hatched lines) are shown as filled symbols. Moment of drug injection (time = 0 min) is shown as vertical hatched line. ***C***, Mean values of three parameters of oxygen response [peak amplitude, s; latency to peak, s; and area under the curve for the total oxygen decrease (AUC)]. Asterisks denote significant between-group differences (*p* < 0.05). Symbols in peak amplitude graph show individual values of oxygen decreases in percentage of baseline.

Fentanyl injected at a 40 μg/kg dose rapidly and strongly decreased NAc oxygen levels (*F*_(5,905)_ = 3.82, *p* < 0.001; Fig. 1*A*). Mean oxygen levels decreased to 35.56 ± 3.76% of baseline (SD = 9.21%), with a maximal effect at 109.3 ± 6.91 s, but rapidly returned to baseline and exhibited a 10–20% oxygen rebound above baseline.

**Figure 2.**
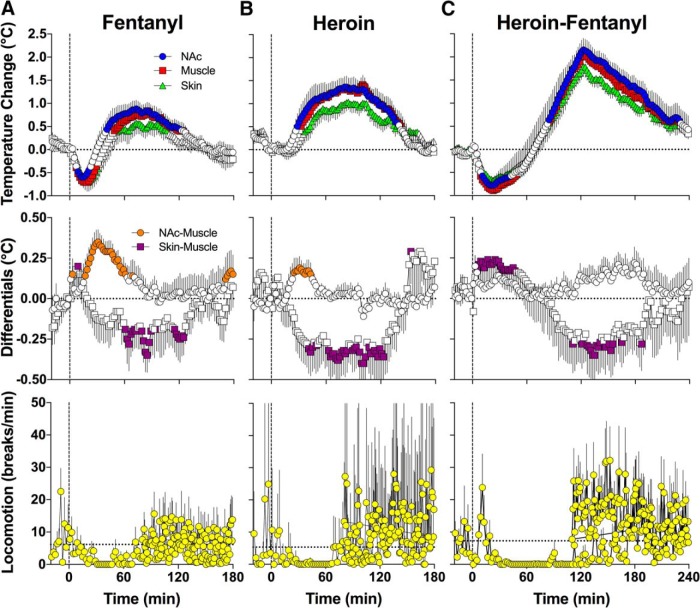
Changes in temperature in the NAc, temporal muscle, and skin (top row), NAc-muscle and skin-muscle temperature differentials (middle row), and locomotor activity (bottom row) induced by intravenous injections of fentanyl (40 µg/kg; ***A***), heroin (400 µg/kg; ***B***), and their mixture (40 µg/kg fentanyl + 360 µg/kg heroin; ***C***) in awake, freely moving rats. Values significantly different from baseline (*p* < 0.05; horizontal hatched lines) are shown as filled symbols. Moment of drug injection (time = 0 min) is shown as vertical hatched line.

Fentanyl induced a biphasic temperature response, with an initial decrease followed by an increase (*F*_(9,810)_ = 7.8, 10.6, and 6.7 for NAc, muscle, and skin temperatures, respectively; *p* < 0.0001; Fig. 2*A*). The brain-muscle differential, a measure of intrabrain heat production, also significantly increased for ∼80 min postinjection (*F*_(9,810)_ = 4.3, *p* < 0.0001). The skin-muscle differential, a measure of skin vascular tone, initially increased slightly but then decreased more strongly (*F*_(9,810)_ = 2.9, *p* < 0.0001). Together, these data indicate that fentanyl at a 40 μg/kg dose causes initial hypothermia due to transient skin vasodilation and heat loss to the external environment, followed by hyperthermia mediated by metabolic brain activation and resultant intrabrain heat production as well as heat retention due to skin vasoconstriction. Fentanyl at this dose decreased locomotor activity and resulted in near-total freezing for 70–80 min postinjection.

### Effects of pure heroin and heroin-fentanyl mixture

Similar to fentanyl, heroin injected at a 400 μg/kg dose significantly decreased NAc oxygen levels (*F*_(7,1680)_ = 6.33, *p* < 0.001; Fig. 1*B*). The effect of heroin was similar in magnitude to that of fentanyl (a decrease to 35.1 ± 4.5% of baseline vs 36.6 ± 3.8%), but the decrease was more prolonged (18 vs 8 min), the maximal decrease occurred at a later time, and the area under the curve was slightly, but significantly larger than that for fentanyl (Fig. 1*C*).

In contrast to fentanyl, heroin monophasically increased temperature in the NAc, muscle, and skin (*F*_(7,630)_ = 8.8, 10.3, and 7.3, respectively; *p* < 0.0001; Fig. 2*B*). The increase showed ∼20 min latency, peaked at ∼90 min, and all temperatures returned to baseline at ∼160 min. Due to larger and more rapid changes in NAc temperature, the NAc-muscle differential significantly increased (*F*_(7,630)_ = 1.9, *p* < 0.001). This effect was relatively transient, delayed, and evident within 15–60 min postinjection. Due to weaker increases in skin temperature, the skin-muscle differential monophasically decreased for ∼150 min postinjection (*F*_(7,630)_ = 4.0, *p* < 0.001). Heroin induced near-total locomotor inhibition for the first 60 min after injection, after which locomotion was slightly elevated above the preinjection baseline.

Similar to fentanyl and heroin injected alone, an opioid mixture containing 360 μg/kg of heroin and 40 μg/kg of fentanyl induced a robust decrease in NAc oxygen levels (*F*_(7,1680)_ = 11.6, *p* < 0.001; Fig. 1*B*). While not significantly different from fentanyl or heroin in its magnitude (Fig. 1*C*), the opioid mixture induced the strongest oxygen decrease (mean 28.69 ± 69% of baseline), with 4/8 cases showing oxygen decreases to lower than 25% of the pre-injection baseline. In contrast to the relatively short-term decrease seen with either fentanyl or heroin alone, the heroin-fentanyl mixture induced a more prolonged decrease, with a return to baseline ∼133 min after the injection. Due to this prolongation of the effect, the area under the curve for the oxygen decrease was ∼10.6-fold larger than for fentanyl alone (*t* = 6.69, *p* < 0.001) and 5.5-fold larger than for heroin alone (*t* = 3.78, *p* < 0.01; Fig. 1*C*). A comparison of the heroin and heroin-fentanyl data using two-way ANOVA revealed a significant interaction (*F*_(240,1680)_ = 4.89) with significant between-group differences from 7.5 to 47.5 min postinjection.

In contrast to pure heroin, the opioid mixture induced a biphasic temperature response evident in all recording locations (*F*_(8,960)_ = 19.2, 21.4, and 20.8 for NAc, muscle, and skin temperatures, respectively; Fig. 2*C*). Temperature initially decreased rapidly and then increased strongly, peaking at ∼120 min and slowly descending toward baseline. The total duration of the temperature response was clearly larger than that for either heroin or fentanyl alone, and temperature at all sites did not fully return to baseline by 4 h postinjection. In contrast to the phasic rise in the NAc-muscle differential seen with pure heroin, the opioid mixture did not elicit significant changes in this parameter (*F*_(8,960)_ = 0.2, *p* > 0.05). The skin-muscle differentials showed biphasic dynamics, with the initial increase followed by a more prolonged and larger decrease. Together, these data indicate that a heroin-fentanyl mixture causes initial hypothermia due to vasodilation and heat loss to the environment, which is followed by robust hyperthermia resulting from intrabrain heat production due to metabolic brain activation. The heroin-fentanyl mixture caused near-total locomotor inhibition from ∼20 to 120 min postinjection, after which locomotor activity increased before returning to its pre-injection baseline ∼200 min postinjection.

### Additional findings resulting from high time-resolution and correlation analyses

**Figure 3.**
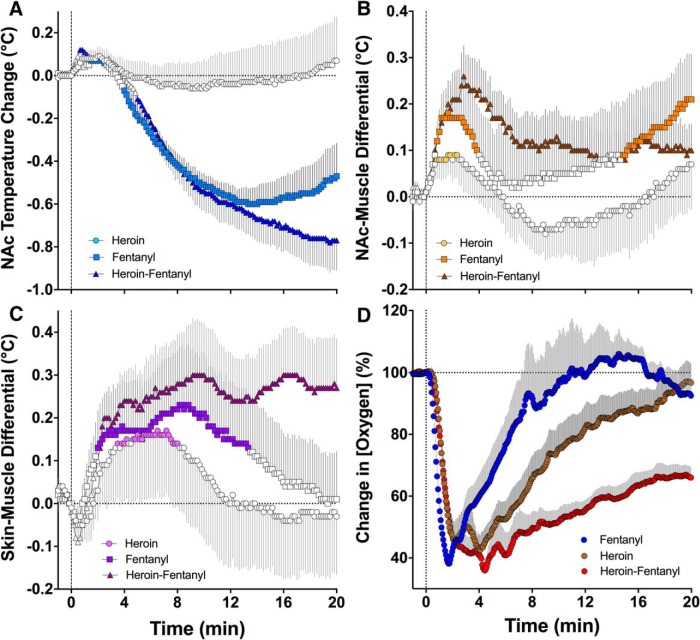
High-resolution analyses of changes in temperature parameters (***A***, NAc temperature; ***B***, NAc-muscle differential; ***C***, skin-muscle differential) and NAc oxygen levels (***D***) for the first 20 min following our treatment (fentanyl, 40 µg/kg; heroin, 400 µg/kg; heroin-fentanyl mixture, 40 µg/kg fentanyl + 360 µg/kg heroin). Each point in all graphs represents mean values for a 10-s bin. Filled symbols in ***A–C*** show values significantly different from baseline (*p* < 0.01).

Since the drug-induced changes in temperature and oxygen were rapid, we also examined the dynamics of these changes with high temporal resolution for the first 20 min postinjection (Fig. 3*A–C*, 10-s bins, *D*, 4-s bins).

Using high-resolution analysis, we found that both fentanyl alone and in combination with heroin induce similarly rapid decreases in NAc temperature, whereas the heroin injection did not produce a temperature decline during this interval (Fig. 3*A*). However, the heroin-fentanyl mixture induced a stronger temperature decrease from ∼13 min postinjection than fentanyl alone. The heroin-fentanyl mixture also produced the strongest and most prolonged increases in NAc-muscle and skin-muscle differentials compared to heroin or fentanyl alone (Fig. 3*B*,*C*). We found that heroin also induces weak but significant increases in NAc-muscle and skin-muscle differentials, suggesting rapid metabolic activation and transient vasodilation as the immediate, transitory effects following its injection. This immediate, transient effect of heroin was not seen when the data were analyzed with low temporal resolution (Fig. 1*B*).

High temporal resolution analysis revealed certain differences in the immediate effects of fentanyl, heroin, and their mixture on NAc oxygen levels (Fig. 3*D*). Each treatment induced comparable decreases in oxygen levels, but the duration of the decrease was shortest with fentanyl, more prolonged with heroin, and longest with the heroin-fentanyl mixture. Importantly, the effects of all treatments were exceptionally rapid, appearing in seconds, well within the injection duration.

**Figure 4.**
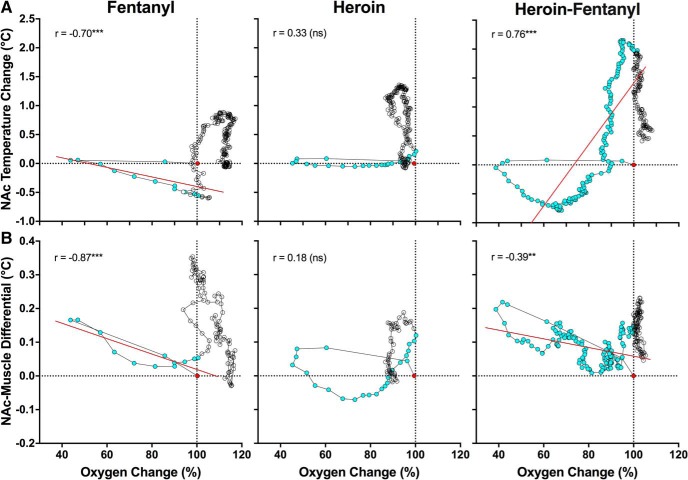
Correlative relationships between drug-induced changes in NAc oxygen and NAc temperature (***A***) and between changes in NAc oxygen and NAc-muscle temperature differentials (***B***). Each graph shows changes in mean values of parameters from the baseline (red point) and span the duration of the temperature responses. Blue symbols show values for the duration of the oxygen response. All graphs show coefficients of correlation calculated for the duration of the oxygen response. Regression lines are shown in red for cases having a significant correlation (**p < 0.01; ***p < 0.001).

To evaluate how heroin-induced changes in oxygen levels are related to brain temperature and the brain-muscle temperature differential, we used time-dependent correlation analyses that show how changes in one parameter are related to changes in the other parameter within the duration of the drug effect (Fig. 4).

As shown in Figure 4A, oxygen levels rapidly decreased and returned to baseline after heroin injection (blue points) but brain temperature remained unchanged. Later, brain temperature increased but this change occurred after brain oxygen had returned to baseline. Therefore, acute hypoxia appears to be the primary effect and the subsequent temperature increase is its potential consequence. Changes in oxygen were also independent of changes in the brain-muscle differential, which subsequently increased presumably as a result of hypoxia (Fig. 4*B*).

Increases in brain temperature also followed decreases in oxygen for fentanyl (Fig. 4*A*), but the relationship was more complex, as a significant negative correlation was found during the period of oxygen decrease (*r* = −0.70, *p* < 0.001; blue points). In this case, temperature remained stable when oxygen dropped but the return of oxygen to baseline was related to decreases in temperature below baseline. A similar negative correlation was found for oxygen and the brain-muscle differential during the period of oxygen response (*r* = −0.87, *p* < 0.001; Fig. 4*B*). However, similar to heroin, the increases in temperature and the brain-muscle differential occurred after the oxygen response, and are perhaps a consequence of the initial hypoxia.

More complex relationships were found with the heroin-fentanyl mixture, which induced a much longer decrease in NAc oxygen (Fig. 4*A*,*B*, blue circles). Similar to the effect seen with heroin and fentanyl alone, oxygen dropped to its maximum level before any changes in temperature were seen, but temperature decreased while oxygen levels slowly returned to baseline. The heroin-fentanyl mixture induced the largest temperature increase, but the increase occurred when oxygen levels were near their baseline. In this case, the oxygen rise correlated with the temperature increase (*r* = 0.76, *p* < 0.001). Despite much more prolonged hypoxia, the relationship between oxygen and the brain-muscle differential after injection of the heroin-fentanyl mixture was similar to that seen with fentanyl alone.

## Discussion

Currently, ∼100 Americans die every day due to opioid overdose (Rudd et al., 2016). To address this national crisis, we developed a model in rats to mimic a human real-world scenario, in which a drug user, who believes he or she is consuming a standard dose of heroin is actually consuming a heroin sample contaminated with a smaller amount of fentanyl, a much more potent opioid drug. It is known that opioid drugs such as heroin and fentanyl induce respiratory depression, which should lead to brain hypoxia. However, it has been impossible until now to directly monitor oxygen levels in the brain’s extracellular space under physiological conditions to examine both physiological fluctuations in this critical metabolic parameter and its drug-induced changes. The technology used in this study allowed us to resolve this limitation and to examine, with second-scale resolution, patterns of changes in NAc oxygen induced by iv heroin, fentanyl, and their mixture in awake, freely-moving rats. While we previously reported that both heroin and fentanyl decrease NAc oxygen levels and showed, by monitoring subcutaneous oxygen levels, that this hypoxic effect results from respiratory depression (Solis et al., 2017b,c), here we aimed to examine how the hypoxic brain effects of heroin at a moderate dose are affected by a minor, 10% contamination with fentanyl. In addition to electrochemical measurements of oxygen changes, we also examined how this drug mixture affects brain temperature as well as two temperature derivatives, NAc-muscle and skin-muscle differentials, which provide measures of intrabrain heat production due to metabolic activation and heat loss due to changes in skin vascular tone, respectively. These data allowed us to examine how oxygen responses induced by opioid drugs are related to their metabolic and vascular effects.

### Fentanyl versus heroin

While it is widely reported that fentanyl is a much more potent drug than heroin, quantitative estimates of potencies depend on the parameter studied and typically vary from 20:1–50:1. In addition, fentanyl is more toxic than heroin, with estimates of LD50 from 5- to 20-fold lower than those for heroin [1–3 mg/kg (von Gunten et al., 2010) vs 15–20 mg/kg (Gable, 2004; Strandberg et al., 2006), respectively]. At the 10:1 dose ratio (40 and 400 μg/kg) used in this study, fentanyl and heroin produced relatively similar decreases in NAc oxygen, suggesting that fentanyl is ∼10-fold stronger than heroin in its ability to induce brain hypoxia.

Both fentanyl and heroin injected at a fraction of their LD50 in rats (2–4% and 2–3%, respectively) decreased oxygen concentrations to 30–40% of baseline, suggesting robust hypoxia. Importantly, these effects were very rapid, appearing during or immediately after the injection and reaching nadir within 100–200 s from the injection onset. For both drugs, oxygen levels recovered relatively quickly, returning to baseline 10–20 min after the injection. However, the fentanyl-induced oxygen drop was more rapid and transient than that for heroin. These between-drug differences in the dynamics of oxygen response could be related to the known differences in pharmacokinetics. While both drugs easily cross the blood-brain barrier and interact with the same μ-opioid receptors, fentanyl directly acts on these receptors, whereas heroin acts as a prodrug, with two pharmacologically active metabolites, 6-MAM and morphine, which display different pharmacokinetics (Boix et al., 2013; Gottås et al., 2013).

Both fentanyl and heroin at a 1:10 dose ratio strongly inhibited locomotor activity with similar durations, but they differed in temperature responses. Both drugs induced modest hyperthermia, but this response for fentanyl was preceded by a temperature decrease evident within the first 30–40 min after the injection. Both drugs also increased NAc-muscle differentials, indicating increased intrabrain heat production as a consequence of metabolic brain activation. This effect, however, was delayed, transient, and stronger for fentanyl than for heroin. Our correlative analysis confirmed that the oxygen drop induced by either fentanyl or heroin is not related to metabolic brain activation, which instead could be an adaptive response caused by hypoxia. Both drugs induced skin vasoconstriction; this effect was the most prolonged of the parameters considered and it could be responsible for the tonic increases in brain temperature induced by each drug. In contrast to heroin, fentanyl initially induced a transient increase in the skin-muscle differential, implying skin vasodilation, which appears to be responsible for the initial decrease in brain temperature seen with this drug.

### Pure heroin versus heroin contaminated by fentanyl

Several important differences in oxygen response were found when rats were injected with heroin with 10% addition of fentanyl. First, the NAc oxygen decrease was greatly potentiated. Although the mean magnitude of oxygen decrease did not differ statistically from heroin alone, in half of the rats the heroin-fentanyl mixture induced decreases lower than 25% of the baseline, suggesting robust hypoxia. Similar decreases were seen only in a quarter of the cases with pure heroin. However, the duration of hypoxia drastically increased after receiving the drug mixture. This change is best represented by the area under the curve, which was over five times larger than that for pure heroin. This prolongation of brain hypoxia is functionally important because brain cells may tolerate transient decreases in oxygen inflow, but are damaged to a greater extent when hypoxia is more prolonged (Hossmann, 1999).

The heroin-fentanyl mixture also induced a distinct temperature response. In contrast to the slow and gradual hyperthermic effect seen with pure heroin, the heroin-fentanyl mixture induced a biphasic brain temperature response, with a prominent decrease for ∼80 min postinjection. This decrease was coupled with an increase in the skin-muscle differential, suggesting skin vasodilation and enhanced heat dissipation as a primary factor determining the decrease in brain and muscle temperatures. Although temperature then increased and its increase was larger than that of heroin alone, the immediate hypothermic effect of the heroin-fentanyl mixture is the most clinically relevant because most acute complications related to fentanyl occur within the first 20 min after its intravenous injection (https://www.erowid.org/).

### Mechanisms underlying brain hypoxia induced by opioid drugs

The brain is one of the heaviest consumers of oxygen in the body and accounts for ∼20% of total oxygen consumption (Siesjo¨, 1978; Rolfe and Brown, 1997). High metabolic activity of the central neurons requires constant and efficient delivery of oxygen that enters the brain extracellular space from the arterial blood via gradient-dependent diffusion (Attwell et al., 2010). Oxygen entry into the brain is enhanced during functional neural activation and this adaptive effect appears to result primarily from neuronal activation that induces local vasodilation and increases local cerebral blood flow (Lecrux and Hamel, 2011; Muoio et al., 2014; Solis et al., 2017a). However, opioid drugs dramatically alter these well-regulated physiological mechanisms by inducing respiratory depression and decreasing blood oxygen levels. Since oxygen diffusion via the blood-brain barrier depends on a concentration gradient, a decrease in blood oxygen levels resulting from respiratory depression is the primary cause of brain hypoxia induced by opioid drugs. This mechanism has been confirmed previously by demonstrating robust oxygen decreases in the subcutaneous space, a highly-vascularized area with no metabolic activity of its own (Solis et al., 2017b,c). Intravenous fentanyl and heroin also induce robust hypotension (Laubie et al., 1974) resulting from decreases in sympathetic output and peripheral vasodilation, a mechanism confirmed by our thermorecording experiments. Therefore, decreased perfusion pressure could also amplify the brain oxygen decreases induced by opioid drugs.

Our correlative analysis confirmed that the oxygen decreases induced by opioid drugs occurred before any increases in brain temperature and brain-muscle differentials (Fig. 4), parameters related to metabolic brain activation. Therefore, respiratory depression and the subsequent drop in brain oxygen levels are the primary neural effects of these drugs, whereas the succeeding hyperthermia and increases in brain-muscle differentials could be an adaptive brain response caused by brain hypoxia. This pattern of drug-induced changes is drastically different from that occurring under normal physiologic conditions, in which intrabrain oxygen inflow elicited by arousing stimuli is tightly related to metabolic neural activation (Solis et al., 2017a).

### Functional and clinical implications

Direct, high-speed evaluation of NAc oxygen dynamics revealed that iv fentanyl, heroin and their mixture induce robust brain hypoxia. Importantly, this effect is remarkably rapid, reaching its maximum at 1.5–4.0 min after the injection onset. While changes in oxygen occurring in humans could have slightly slower dynamics than in rats, the exceptionally rapid time course of the brain hypoxic response poses significant constraints on using opioid antagonists such as naloxone to treat this dangerous condition. While naloxone is highly effective in blocking most effects of opioid drugs, to be effective it should be delivered within a critical time window following opioid administration. This issue could be less important for subcutaneous injections or oral ingestion of these drugs since these routes of drug delivery possess much slower drug pharmacokinetics and a longer time window for possible treatment.

As shown in this study, even minor contamination of heroin with fentanyl results in strong potentiation of brain hypoxia. In contrast to the relatively transient brain oxygen decreases seen with fentanyl and heroin alone, the hypoxic response became sustained with the heroin-fentanyl mixture, persisting for up to 2 h after drug administration. In addition, the heroin-fentanyl mixture prolonged motor inhibition and strongly decreased brain and body temperature for ∼1 h due to initial vasodilation. This response pattern, suggesting deep sedation, is typical for general anesthesia (Kiyatkin and Brown, 2005) and could underscore another important feature of fentanyl: its ability to induce an acute state of unconsciousness, fully blocking motor responses to external and internal stimuli (Jaffe et al., 1997). Therefore, the inability to control one’s body, particularly to maintain respiratory function coupled with strong respiratory depression, could increase the likelihood of asphyxiation. The combination of these two dangerous effects could result in death when fentanyl is injected at a high dose by mistake instead of heroin or when a heroin sample is contaminated with fentanyl.

## Synthesis

Reviewing Editor: Kirill Martemyanov, The Scripps Research Institute Decisions are customarily a result of the Reviewing Editor and the peer reviewers coming together and discussing their recommendations until a consensus is reached. When revisions are invited, a fact-based synthesis statement explaining their decision and outlining what is needed to prepare a revision will be listed below. Note: If this manuscript was transferred from JNeurosci and a decision was made to accept the manuscript without peer review, a brief statement to this effect will instead be what is listed below.

I have reviewed your responses to the original reviewers at Journal of Neuroscience and found changes that you made to be satisfactory.
